# Corn or Soybean Oil as the Sole Carbon Source for Polyhydroxybutyrate Production in a Biofuel Biorefinery Concept

**DOI:** 10.3390/polym17030324

**Published:** 2025-01-25

**Authors:** Clara Matte Borges Machado, Luciana Porto de Souza Vandenberghe, Ariane Fátima Murawski de Mello, Carlos Ricardo Soccol

**Affiliations:** Department of Bioprocess Engineering and Biotechnology, Centro Politécnico, Federal University of Paraná, Curitiba 81531-980, PR, Brazil; claramachado@ufpr.br (C.M.B.M.); ariane.mello@ufpr.br (A.F.M.d.M.); soccol@ufpr.br (C.R.S.)

**Keywords:** alternative substrates, bioplastics, PHB, polyhydroxyalkanoates, vegetable oils

## Abstract

The use of polyhydroxybutyrate (PHB) can help diminish fossil chemical dependency because it can partially replace petrochemical plastics due to its biodegradability and similar mechanical properties. However, its production costs are high compared with fossil-based plastics. Alternative carbon sources can be used in the fermentation media because they are renewable and low-cost. Vegetable oils are especially attractive due to their high carbon content, contributing to high production rates per gram of substrate. This work aimed to produce PHB from *Cupriavidus necator* LPB1421 using either corn or soybean oil as the sole carbon source. Urea was the best nitrogen source, enabling a DCW production of 4.35 g/L (corn oil) and 10.4 g/L (soybean oil). After media optimization, the DCW of corn oil reached 22.13 g/L, with 57.46% PHB accumulation (12.71 g PHB/L), whereas soybean oil led to a DCW of 19.83 g/L, with 54.91% PHB accumulation (10.89 g PHB/L). This media composition was employed in a kinetics assay, revealing similar fermentation parameters among both oils and a yield of 0.2118 g PHB/g for corn oil and 0.1815 g PHB/g for soybean oil. These results open the possibility of integrating PHB production with biofuel manufacturing in a bioethanol/biodiesel biorefinery concept.

## 1. Introduction

There is worldwide concern to avoid and substitute the use of fossil-based chemicals because they are non-renewable, scarce, and can release toxic chemicals into the atmosphere, contributing to global warming and environmental pollution. Aiming to reduce the dependency on fossil chemicals, renewable vegetable sources have been proposed to produce biodiesel, bioethanol, biochemicals, and bioplastics [1,2]. Biofuels are produced mostly from corn, wheat, soybeans, vegetable oils, and sugarcane [3]. World corn and bioethanol production is led by the United States of America, responsible for 50% of the worldwide production of bioethanol from maize crops [4].

In the Brazilian context, corn and soybean are important commodities. It is estimated that in the 2023/2024 harvest, the corn production could reach 110.96 million tons, and soybean production is expected to be 147.35 million tons [5,6]. Brazil is responsible for 30% of global bioethanol production, which is mainly obtained from sugarcane fermentation [7]. Additionally, Brazil has nine industrial plants producing bioethanol from corn, allowing flexible industries that can produce it either from corn and/or sugarcane, depending on the season and availability [8]. Regarding biodiesel, Brazil is responsible for 39% of global soybean production and it is the fourth greatest biodiesel producer from soybean oil [4].

Other than biofuel production, corn and soybean crops can be used in polyhydroxybutyrate (PHB) production [9,10], which is especially interesting in the Brazilian context. PHB is a biopolyester produced by microorganisms and it is an interesting alternative to petrochemical plastics due to its biodegradability and biocompatibility [11]. Under unbalanced growth conditions like high carbon sources and limited nutrients or high carbon-to-nitrogen (C:N) ratios, those polymers accumulate in the cytoplasm as energy storage and can represent up to 90–97% of the dry cell weight [12]. Additionally, PHB has similar elastomeric and thermoplastic properties compared with traditional chemical plastics and is especially attractive as a substitute for traditional plastics [13]. Recent calculations suggest that PHB can only substitute ~2% of the total current plastic production [14]. However, this estimation considered PHB production exclusively from sugarcane xylose, and it is known that PHB can be obtained from different renewable resources such as milled corn [9], soybean hulls [10], cheese whey [15], and corn oil [16]. Therefore, to successfully have a positive environmental impact, it is necessary to consider PHB production using a wide range of substrates. Also, the replacement of traditional plastics with biodegradable ones can help reduce fossil chemical dependence, but the high price of PHB is not yet competitive with those of traditional plastics.

It is estimated that the production costs of synthetic plastics are around USD 1250/Mt and those of polyhydroxyalkanoates (PHAs, the family of PHB) are between USD 4000 and USD 15,000 per Mt [17]. This is mainly driven by highly priced carbon sources, representing up to 40% of the product cost [18]. Therefore, it is necessary to establish a process that requires inexpensive carbon feedstocks, low-cost operations, and high production rates [13].

A strategy to reduce costs is the production of PHB in a biorefinery context. This allows the use of the same physical space and equipment to obtain different molecules; also, there is easy access to the same feedstock and the possibility of reusing process by-products, decreasing production and transportation costs [19]. Biopolymer production in biorefineries can also provide a solution for waste management, reducing dependency on non-renewables as well as carbon footprints [20]. In this strategy, the use of renewable feedstocks as the carbon source can contribute even more to the cost reduction.

In this sense, corn and soybean oils are especially interesting for use in PHB production. In corn bioethanol production, corn oil is generated as a by-product [21] and it is estimated that for every ton of corn, 18 L of corn oil is produced [19]. An integrated production of bioethanol and PHB could contribute to a mitigation of production costs in a sustainable manner. When glucose was substituted with corn oil for PHB production from *Acinetobacter baumannii* P39, a 23% cost reduction in the total input cost was observed [16]. This indicates the feasibility of producing PHB in a biorefinery concept with reduced costs.

Regarding soybean oil, it is mostly used in biodiesel production, especially in Brazil, and it is produced using a transesterification process [2]. The coupled production of PHB and biodiesel from soybean oil could diversify a production plant and enhance overall productivity levels. Another advantage of vegetable oils is their higher carbon content in comparison with sugars, which provides a high yield of produced PHB per g of carbon source [22].

There are already a couple of reports in the literature about PHB production from vegetable oils. For example, Volova and collaborators (2020) [23] analyzed different types of oils as substrates such as palm, Siberian oilseed, and sunflower, revealing that the biopolymer molecular weight depends on the fatty acid composition of each oil. Furthermore, as oils are very heterogeneous substrates, genetic engineering could be employed to enhance the strain substrate range, tolerance, and robustness [19]. However, due to specific regulatory policies regarding the use of genetically engineered microorganisms, their use could be burdensome.

Therefore, the main objective of this work was to use a non-recombinant strain (*C. necator* LPB1421) to produce PHB from corn and soybean oils. For that, a strategy for strain adaptation was adopted to assure bacterial growth and PHB production from the process. Different nitrogen sources were analyzed and the one contributing to higher biomass production was selected. Statistical optimization was employed to determine the best concentration of oil, nitrogen, and phosphorous source for PHB production. The best condition was validated and employed in a kinetic study. Finaly, it was proposed that the use of corn and soybean oils could enable the production of this biopolymer within a biorefinery context.

## 2. Materials and Methods

### 2.1. Strain and Reagents

Corn and soybean oils were purchased from a local supermarket (Curitiba, Paraná, Brazil). Standard polyhydroxybutyrate was purchased from Sigma Aldrich^®^ (Darmstadt, Germany). The *Cupriavidus necator* LPB1421 strain was selected from the microbial bank at the Bioprocess Engineering and Biotechnology department of the Federal University of Paraná (Curitiba, Paraná, Brazil).

### 2.2. Strain Adaptation

The *C. necator* LPB1421 strain was successively cultivated in inoculum media (25 mL, 24 h, 30 °C, and 120 rpm), gradually increasing the oil concentrations and decreasing the glucose levels [24]. Six successive cultivations were performed, and each culture was the inoculum to the next one. The first cultivation had only glucose as the carbon source; the second had a glucose–oil ratio of 80:20, the third was 60:40, the fourth was 40:60, the fifth was 20:80, and the sixth was 0:100. In the last step, when there was only the vegetal oil (soybean or corn) as the carbon source, the cells were decanted (48 h), transferred to the conservation media (48 h; 30 °C) and stored (4 °C) until further use. 

### 2.3. Inoculum and Conservation Media

The inoculum media were composed of corn or soybean oil as the carbon source (20 g/L), yeast extract (10 g/L), and peptone (5 g/L) [24]. The inoculum cultivation of oil-adapted cells was carried out for 48 h at 30 °C and 120 rpm in 125 mL Erlenmeyer flasks, with 25 mL of the media in an orbital shaker (SL-223, Solab, Piracicaba, São Paulo, Brazil) inoculated with three loopfuls of bacterial cells. Strains were preserved on Petri dishes using the same media compositions added to agar (15 g/L).

### 2.4. Screening of Nitrogen Source for Biomass Production

To select the best nitrogen source for biomass production, cultivation was carried out in standard fermentation media, varying the type of nitrogen source. The fermentation media were composed of either corn or soybean oil as the carbon source (20 g/L), (NH_4_)_2_SO_4_ (4 g/L), yeast extract (1 g/L), KH_2_PO_4_ (13.3 g/L), MgSO_4_.7H_2_O (1.2 g/L), citric acid (1.7 g/L), and a trace element solution (TES; 10 mL/L), which consisted of FeSO_4_ (10 g/L), ZnSO_4_ (2.25 g/L), CuSO_4_ (1 g/L), CaCl_2_ (2 g/L), Na_2_B_4_O_7_ (0.23 g/L), MnSO_4_ (0.5 g/L), (NH_4_)_6_Mo_7_O_24_ (0.1 g/L), and 35% HCl (10 mL/L) [25]. To select the best nitrogen source for *C. necator* growth, (NH_4_)_2_SO_4_ (4 g/L) from the standard fermentation media was replaced with urea (CH_4_N_2_O), ammonium phosphate ((NH_4_)_3_PO_4_), or ammonium chloride (NH_4_Cl). The pH was set to 6–7 with the addition of KOH and sterilized in an autoclave. Salt solutions were separately autoclaved to avoid precipitation, and the TES solution was sterilized via filtration. The inoculum (10% *v*/*v*) and the other solutions were added under sterile conditions. Fermentation was carried out in 250 mL Erlenmeyer flasks with 50 mL of the media in an orbital shaker for 96 h at 30 °C and 150 rpm.

### 2.5. Optimization of Medium Composition for PHB Production

To study how the vegetable oils, urea, and KH_2_PO_4_ interacted and contributed to bacterial growth and PHB production, a response surface methodology (RSM) with a 2^3^ central composite design (CCD) was applied, totaling 16 assays. Independent variables (either corn or soybean oil as the carbon source, urea, and KH_2_PO_4_ concentration) and their levels were set based on reported results [9]. Data were collected and analyzed using Statistica^®^ 5.0 software (Statista Inc., New York, NY, USA).

### 2.6. Kinetics of PHB Production

The kinetics of cell growth, PHB production, and accumulation were carried out in the optimized media with either corn or soybean oil as the carbon source (60 g/L), urea (2.5 g/L), KH_2_PO_4_ (5 g/L), citric acid (1.7 g/L), TES (10 mL/L), and 10% (*v*/*v*) of the inoculum ratio. The same conditions described above were used and samples were collected every 12 h.

### 2.7. Analytical Methods

Cell growth was quantified by means of the dry cell weight (DCW) using a gravimetric method. In total, 2 mL of the culture broth was transferred to a pre-weighed Eppendorf tube, harvested by centrifugation (4000 rpm; 15 min), and washed twice with distilled water. The tube containing the cells’ pellet was dried overnight (105 °C), transferred to a desiccator until it reached room temperature, and weighed using an analytical balance.

PHB accumulation and production were assessed using gas chromatography (GC-2010, Shimadzu, Kyoto, Japan) after derivatization, following an acid methanolysis protocol [9,10,25]. Briefly, 40 mL of the fermented broth was centrifuged (4000 rpm; 15 min) and washed twice with distilled water. The pellet was then resuspended in 2 mL distilled water, stored overnight (−80 °C), and lyophilized. Lyophilized cells (10 mg) were weighed in borosilicate tubes and 2 mL methanol acid (3% *v*/*v* H_2_SO_4_) was added, followed by the addition of 2 mL chloroform (HPLC grade). The tubes were vortexed (30 s) and incubated in a thermoreactor (140 min and 100 °C; Thermoreaktor TR300, Merck, Darmstadt, Germany). Every 30 min, the tubes were vortexed to assure sample homogeneity. After 140 min, the tubes were cooled to room temperature and 1 mL Na_2_CO_3_ (60 g/L) was added to each tube. The tubes were centrifuged (3000 rpm; 10 min), the top aqueous layer was discarded, and 5 mg Na_2_SO_4_ was added to remove any residual water. The lower organic layer was filtered through a 0.22 μm hydrophobic membrane filter and the sample was analyzed using GC-2010 apparatus, as previously described [9,10]. Benzoic acid was employed as the internal standard and poly((R)-3-hydroxybutyric acid) (Sigma-Aldrich, Burlington, MA, USA) was the external standard.

## 3. Results and Discussion

### 3.1. Screening of Nitrogen Sources for C. necator Growth

The type of nitrogen source and its concentration are extremely important for bacterial growth. The substitution of ammonium sulphate (reference medium) by urea represented a significant DCW increase from 2.27 ± 0.60 g/L to 4.35 ± 0.41 g/L with corn oil and 4.4 ± 0.56 g/L to 10.4 ± 0.37 g/L with soybean oil as carbon sources. Overall, urea was shown to be the best source for *C. necator* cell growth (Table 1). This could be linked to its chemical composition because it was the only nitrogen source with carbon in its structure, contributing to biomass development.

The use of urea as a nitrogen source is advantageous because it is easily transported into bacterial cells, it does not dissociate in ions, and it is not pH-dependent as inorganic nitrogen sources are [9]. Furthermore, urea is cheap [15], helping to reduce PHB production costs. Other studies have already reported good bacterial growth with urea as the nitrogen source and using different carbon sources such as fructose for *Ralstonia eutropha* [26], sucrose for *Azohydromonas lata* MTCC 2311 [27], and jatropha and waste cooking oil for *Cupriavidus necator* H16 [28,29].

### 3.2. Optimization of Medium Composition for PHB Production

As stated previously, PHB accumulation is dependent on unbalanced growth conditions, especially high carbon and low nutrient concentrations. The interaction between carbon, nitrogen, and phosphorous sources can greatly impact biopolymer production [9]. Additionally, phosphorous deprivation blocks DNA synthesis and cell division, contributing to metabolic stress and PHB synthesis [18]. Therefore, a response surface methodology (RSM) with a 2^3^ central composite design (CCD) was applied to observe the interaction between either corn or soybean oil, urea, and KH_2_PO_4_ sources as well as their concentrations in the media. The DCW as well as PHB accumulation and production were the analyzed responses (Table 2).

**Figure 1:**
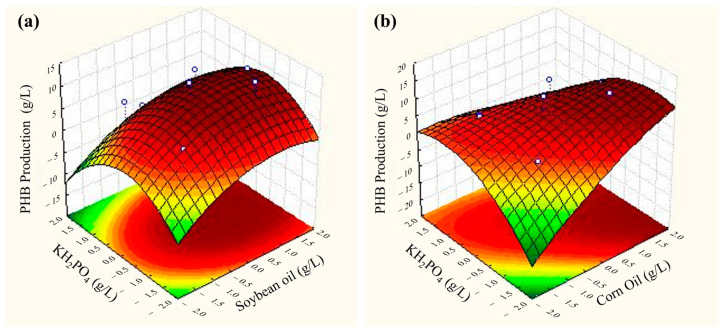
CCD response surface plots of phosphorous and carbon source interactions for a PHB production model with (**a**) soybean oil and (**b**) corn oil.

As expected, higher carbon concentrations with low urea and KH_2_PO_4_ contents contributed to high PHB and biomass production (Figure 1). For example, assay 9 (negative carbon source axial point) showed a low DCW and PHB accumulation and production for both substrates. Contrarily, assay 5 was the best condition for both substrates, resulting in a DCW of 20.70 g/L, with 62.24% PHB accumulation (12.88 g/L or 0.214 g/g substrate) for soybean oil. In the case of corn oil, the obtained DCW was 23.05 g/L, with 61.28% accumulation, equivalent to 14.13 g/L PHB or 0.214 g/g substrate. This condition was then selected and validated using soybean oil (DCW of 18.45 g/L, with 57.98% accumulation and 10.70 g/L PHB production) and corn oil (DCW of 23 g/L, with 58.46% accumulation and 13.44 g/L PHB production). In this condition (5), the C:P:N ratio was 24:2:1, showing that unbalanced nutrient- and carbon-source concentrations favored PHB production and accumulation. Therefore, the concentrations of the carbon source, urea, and KH_2_PO_4_ used in assay 5 were used for further PHB production.

All the models showed satisfactory results, with R^2^ values above 0.7 (Table 3); these were adjusted by removing interactions with no statistical significance (*p* > 0.05), which were considered for the analysis. Overall, the soybean models had a lower R^2^ value when compared with the corn-oil models, possibly because the responses observed with soybean oil presented fewer variations than with corn oil. In the adjusted models, the carbon and phosphorous sources were significant (*p* < 0.05). For both substrates, the PHB production model was the best fitting for the process. With soybean oil, the linear carbon source and quadratic phosphorous source were significant. As for corn oil, the following variables showed a *p*-value < 0.05: linear carbon source, quadratic and linear nitrogen source, quadratic phosphorous source, interaction between carbon and nitrogen source, and interaction between carbon and phosphorous source. All the Pareto charts regarding these analyses are available in the Supplementary Materials.

Similarly, Kynadi and Suchithra [30] also employed an RSM to enhance PHB production from *Bacillus cereus*. In this case, rubber-seed oil, the agitation speed, and the fermentation time were the optimized parameters. It was found that higher oil concentrations led to a higher PHB content (above 640 mg/g DCW). Also, Prasanth and collaborators (2022) [31] used the RSM strategy to optimize PHBV production from *C. necator* using *Madhuca indica* oil (mahua-tree oil). The inoculum-, nitrogen-, and carbon-source concentrations were optimized. The authors also found that a higher carbon content and an intermediate nitrogen-source concentration led to a higher PHBV production (5.81 g/L). This shows that the results presented in this work exceeded those reported in the literature regarding PHB production optimization using vegetable oil as the sole carbon source. Also, the process developed here differed from those already published in the literature. In this study, no treatment of the vegetable oils was performed prior to the fermentation and no emulsifier was added to the media, as reported in other studies in the literature [23,32]. This eliminates unit operations, facilitating PHB production and reducing costs.

### 3.3. Kinetics of PHB Production Using Corn and Soybean Oils

**Figure 2:**
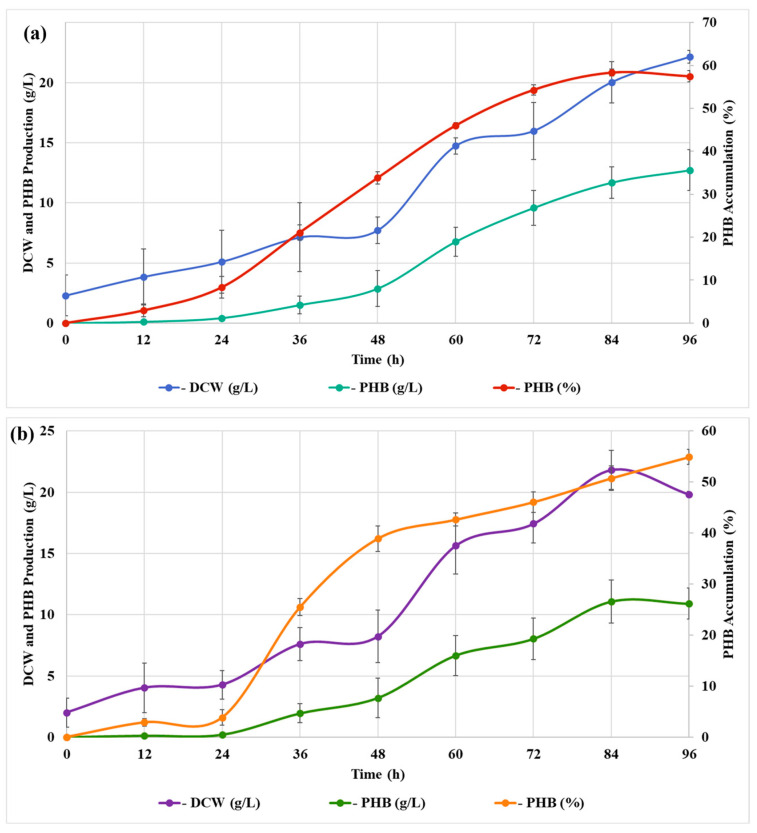
Fermentation kinetics of DCW production and PHB production and accumulation with (**a**) corn oil and (**b**) soybean oil.

The optimized media (60 g/L carbon source, 2.5 g/L urea, and 5 g/L KH_2_PO_4_) were employed in the fermentation kinetics assay because they were the best conditions for both oils. The exponential phase for biomass production occurred between 24 h and 84 h for both substrates (Figure 2), with a slight DCW decrease at 96 h for soybean oil (Figure 2b). Maximum DCW productivity was reached at 60 h for corn oil (0.245 g/L.h) and soybean oil (0.260 g/L.h). In both substrates, PHB production and accumulation had an exponential phase beginning at 24 h, reaching a stationary phase at 72 h of cultivation, indicating that the available carbon source (either corn or soybean oil) was completely or almost totally consumed at this point. PHB production showed maximum productivity at 84 h for corn oil (0.139 g/L.h) and soybean oil (0.132 g/L.h). A similar PHB accumulation profile was found by Kamilah et al. (2013) [28] when cultivating recombinant *C. necator* in fresh and waste cooking oil, achieving a maximum PHA accumulation (85%) at 72 h of cultivation (0.263 g/L.h).

The specific growth rate was similar for both oils (Table 4), and was 0.0236 h^−1^ for corn oil and 0.0239 h^−1^ for soybean oil. The DCW and product yield were slightly higher for corn oil (0.3688 g DCW/g oil; 0.2118 g PHB/g oil) than for soybean oil (0.3305 g DCW/g oil; 0.1815 g PHB/g oil).

**Figure 3:**
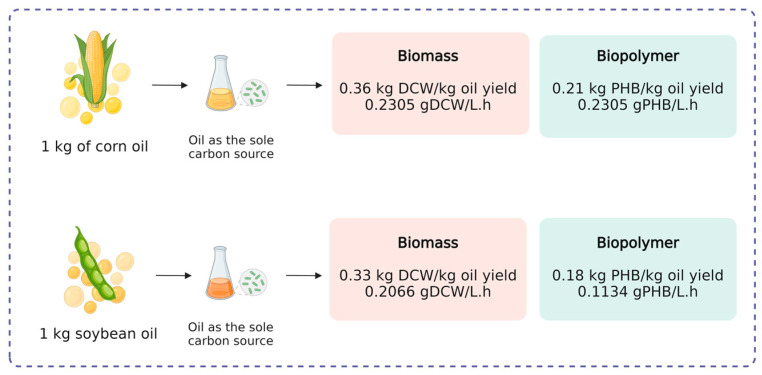
Mass balance of biomass and PHB production from 1 kg of corn or soybean oil.

The higher yields obtained for corn oil were in accordance with the kinetics data, where higher PHB production levels were achieved with corn oil. This could be related to the fatty acid profile of each oil. Corn oil is composed of linoleic acid (58.7%), oleic acid (26.6%), palmitic acid (11.5%), stearic acid (2.2%), linolenic acid (0.8%), and arachidic (eicosanoic) acid (0.2%) [33], whereas soybean oil is composed of linoleic acid (55%), oleic acid (18%), linolenic acid (13%), palmitic acid (10%), and stearic acid (4%) [34]. The main difference between both oils is the amount of oleic and linolenic acids. Corn oil presents more oleic acid and less linoleic acid than soybean oil, indicating that the *C. necator* LPB1421 strain could grow and produce higher PHB titers in an environment with lower concentrations of linolenic acid and greater concentrations of oleic acid. Additionally, a slight decrease in the DCW and PHB production was observed at the end of the fermentation with soybean oil (Figure 2), which could also be linked to the lower yields of this substrate in comparison with corn oil. Furthermore, these data suggest that it is possible to obtain 211.8 g PHB from 1 kg of corn oil and 181.5 g PHB from 1 kg of soybean oil (Figure 3).

These results were similar to those reported in the literature. For example, when cultivating *C. necator* DSM 545 in spent coffee-grounds oil, Ingram and Winterburn [35] reported a yield of 0.53 g DCW/g oil and PHA productivity of 0.11 g/L.h. Similarly, for sunflower oil, a biomass yield of 0.56 g DCW/g oil and PHA productivity of 0.12 g/L.h were obtained. Additionally, Peña-Jurado and collaborators (2019) [15] cultivated *Bacillus subtilis* EPAH18 in cheese whey and reported a product yield of 0.057 g PHB/g substrate. These results could indicate that the fermentation parameters are greatly influenced by the selected strain as well as the chosen substrate.

Different substrates lead to different types of PHAs [36]. For example, when a sugar-rich substance is used, the glucose is converted to Acetyl-CoA→Acetoacetyl-CoA→3-Hydroxybutyryl-CoA, which is then converted to PHB by SCL PHA synthase. However, when oil-rich substrates are employed, the fatty acids undergo either in situ fatty acid synthesis or the β-oxidation pathway. In both cases, R-3-Hydroxyacyl-CoA is converted into mcl-PHA by MCL PHA synthase. Other than the type of substrate, the final produced PHA also depends on the strain. Obruca and colleagues (2014) [37] reported a PHB production method using an oil-rich substrate (spent coffee grounds) for *C. necator* H16. The same strain was also used by Ng and collaborators (2010) [29] using another vegetable oil (jatropha oil) and the final polymer was also reported to be PHB. On the other hand, when *C. necator* DSM 545 was cultivated in either spent coffee-grounds oil or sunflower oil, Ingram and Winterburn [35] reported the production of a PHA co-polymer, P(3HB-coHV). In the case of this study, only the production of HB monomers was observed when using *C. necator* LPB 1421 cultivated with corn or soybean oil as the substrate. The results reported in the literature further strengthen the results obtained in the study presented here. That is, the type of PHA produced depends on both the type of employed substrate and the chosen bacterial strain.

### 3.4. Perspectives on the Implementation of the Proposed Process in a Biofuel Biorefinery Context

World corn production for the 2023/2024 season is expected to reach 1232.6 million tons, a 6.6% increase compared with the previous season. Soybean production worldwide is also expected to increase in the 2023/2024 season, reaching 398.2 million tons—a 5.3% increase compared with the last season [38]. Brazil and the USA are the major players in both soybean and corn production, and are in the top three countries worldwide that most produce both commodities.

Usually, these two crops are destined for biofuel production, generating several by-products. For example, from 1 ton of milled corn, 400 L bioethanol can be obtained, generating 268 kg distiller’s dried grains with solubles (DDGSs), 16 L corn oil, and 288 kg CO_2_ [39]. Regarding soybeans, biodiesel can be produced through the transesterification of the soybean oil. To obtain this oil, the feedstock needs to be processed. It is estimated that from 1 ton of soybean, 182.95 kg oil and 799.78 kg protein-rich meal are produced. With the data presented above about the expected world production of soybeans and corn and the amount of oil generated from 1 ton of each feedstock, it is possible to estimate that 19,721.6 million tons of corn oil and 72,850.69 million tons of soybean oil will be produced in the 2023/2024 season. Considering the PHB yield (in g/g) that can be obtained from corn and soybean oils (Figure 3) and using Fermi calculations [14], it is possible to estimate that, from the 2023/2024 season, 3.93 million tons of PHB could be produced from corn oil and 13.11 million tons of PHB could be synthetized from soybean oil. This amount of PHB could replace 4.26% of the world’s petrochemical annual plastic production (~400 million tons). However, it is important to point out that not all vegetable oil produced is destined for PHB production because it can be used as food-grade chemicals or even as a substrate for biodiesel synthesis. Also, to effectively substitute petrochemical plastics for PHB, this biomaterial should be produced from a wide range of renewable sources such as lignocellulosic residue, waste biomass, and even wastewater.

**Figure 4:**
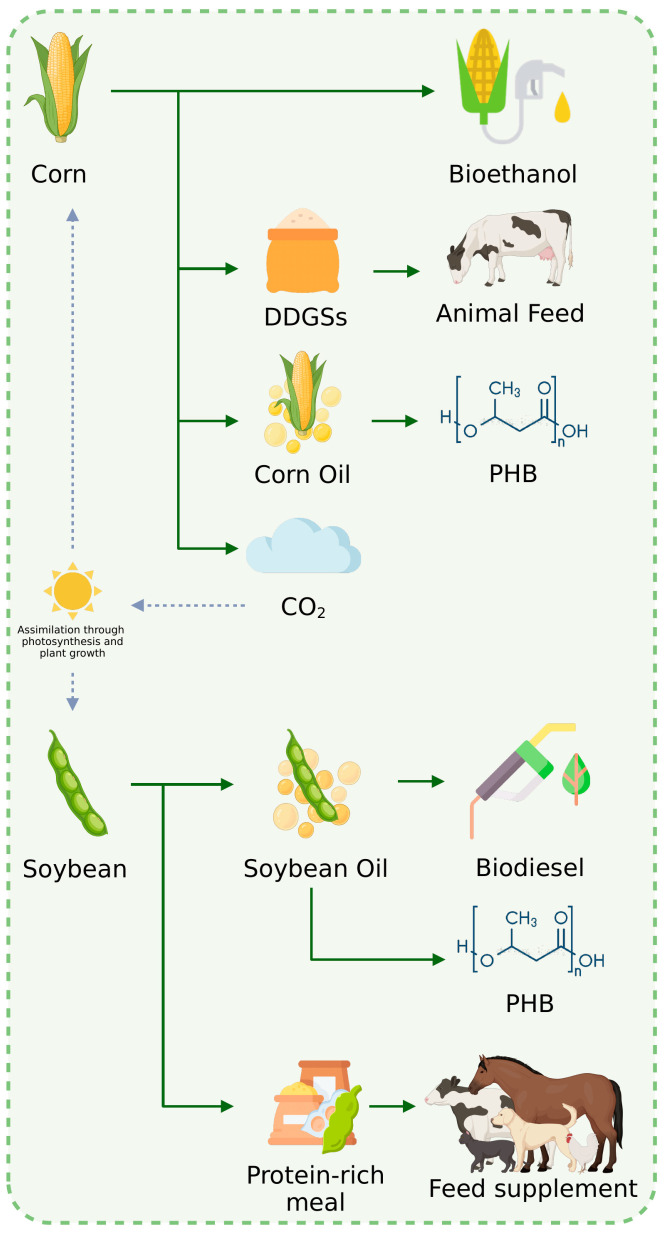
PHB production from oil-rich substrates within a corn–soybean biofuel biorefinery.

Nevertheless, the generation of such expressive quantities of oil-rich substrates during corn and soybean processing alongside the results reported here enables the production of PHB within a biofuel biorefinery context (Figure 4). There is already an industry that produces PHAs in a biorefinery, the BluePHA company, which produces PHAs from plant oils or starch and bio-fixed CO_2_ in a closed loop [40]. It has the capacity to produce 5000 tons of PHA annually; this is expected to grow to 25,000 tons when new facilities are implemented.

This is an actual example of PHA production in a biorefinery from oil-rich substrates, showing that it is possible to integrate the production of the biopolymer into a biofuel production platform. However, to achieve that, further studies on process scale-up are necessary. Also, to effectively produce PHB in a biorefinery, some challenges need to be overcome such as transporting the oils generated from various industries to the desired location; accommodating different substrate compositions in the same process, thus assuring reproducibility; and developing cost-effective downstream processes [41].

## 4. Conclusions

A *Cupriavidus necator* non-recombinant strain was able to grow and produce PHB using corn or soybean oil as the sole carbon source. Urea was found to be the best nitrogen source for biomass production in both substrates. It was confirmed that high carbon and low nutrient contents (C:P:N ratio of 24:2:1) contributed to higher biomass and PHB production titers. Similar fermentation parameters were achieved using both substrates, with a biopolymer yield of 0.2118 g PHB/g corn oil (0.1324 g/L.h) and 0.1815 g PHB/g soybean oil (0.1134 g/L.h). The use of vegetable oils as the sole carbon source for PHB production can present some drawbacks such as difficulties regarding the use of oils at an industrial scale, especially when cleaning equipment using residual oils. Further process improvements could be achieved by studying different operation modes and scaling-up strategies. The results presented here confirm the possibility of integrating PHB, biodiesel, and bioethanol production. To the best of our knowledge, this is the first work that reports PHB production from corn and soybean oils using a non-recombinant strain in a biofuel biorefinery concept.

## Figures and Tables

**Table 1:** Influence of different nitrogen sources on *C. necator* DCW production.

Nitrogen Source	DCW (g/L)	
	Corn Oil	Soybean Oil
Urea (CH₄N₂O)	4.35 ± 0.41	10.4 ± 0.37
Ammonium chloride (NH_4_Cl)	2.17 ± 0.39	5.68 ± 0.67
Ammonium phosphate ((NH_4_)_3_PO_4_)	2.5 ± 0.35	2.88 ± 0.25
Ammonium sulphate (NH_4_)_2_SO_4_	2.27 ± 0.60	4.4 ± 0.56

**Table 2:** CCD conditions and results.

				Soybean Oil				Corn Oil			
Assay	Oil (g/L)	Urea (g/L)	KH_2_PO_4_ (g/L)	DCW (g/L)	Accumulation (%)	PHB (g/L)	PHB (g/g)	DCW (g/L)	Accumulation (%)	PHB (g/L)	PHB (g/g)
1	20	2.5	5	11.83	39.37	4.66	0.2330	3.50	33.34	0.12	0.0060
2	20	2.5	15	11.95	59.11	7.06	0.3530	12.63	47.55	6.00	0.3000
3	20	7.5	5	10.90	46.46	5.06	0.2530	9.15	42.14	3.86	0.1930
4	20	7.5	15	3.25	9.78	0.32	0.0160	10.03	58.44	5.86	0.2930
5	60	2.5	5	20.70	62.24	12.88	0.2147	23.05	61.28	14.13	0.2355
6	60	2.5	15	6.25	32.74	2.05	0.0342	14.48	54.70	7.92	0.1320
7	60	7.5	5	12.05	58.01	6.99	0.1165	12.60	56.32	7.10	0.1183
8	60	7.5	15	14.58	54.23	7.90	0.1317	8.80	39.78	3.50	0.0583
9	6.4	5	10	6.80	9.50	0.65	0.1016	6.65	23.62	1.57	0.2453
10	73.6	5	10	17.83	55.49	9.89	0.1344	15.28	59.79	9.13	0.1240
11	40	0.8	10	15.88	75.43	11.98	0.2995	14.60	84.15	12.29	0.3073
12	40	9.2	10	15.05	48.31	7.27	0.1818	13.40	58.93	7.90	0.1975
13	40	5	1.6	10.53	26.95	2.84	0.0710	11.03	29.04	3.20	0.0800
14	40	5	18.4	3.88	8.94	0.35	0.0088	4.08	7.40	0.30	0.0075
15	40	5	10	16.08	55.76	8.96	0.2240	15.88	50.25	7.98	0.1995
16	40	5	10	15.15	59.07	8.95	0.2238	14.43	50.13	7.23	0.1808

**Table 3:** CCD models and parameters.

	Model	R^2^	R^2^ Adj.	Error	MS Residual
Soybean Oil	DCW	0.76749	0.41872	86.781	14.46351
	DCW Adjusted	0.7036	0.506	110.6276	12.29195
	PHB Accumulation	0.78773	0.46932	1358.649	226.442
	PHB Accumulation Adj	0.69496	0.49161	1952.386	216.932
	PHB Production	0.78129	0.45323	53.3403	8.89006
	PHB Production Adj	0.72066	0.53443	68.1286	7.56985
Corn Oil	DCW	0.87877	0.69692	43.1419	7.1903
	DCW Adjusted	0.87452	0.73111	44.6557	6.3794
	PHB Accumulation	0.86101	0.65252	912.961	152.16
	PHB Accumulation Adj	0.8337	0.64365	1092.304	156.043
	PHB Production	0.96645	0.91613	7.8675	1.31124
	PHB Production Adj	0.96559	0.92627	8.0691	1.15272

**Table 4:** *C. necator* LPB 1421 fermentation parameters for corn and soybean oils.

Fermentation Parameter	Corn Oil	Soybean Oil
Specific Growth Rate, μ (h^−1^)	0.0236	0.0239
DCW Productivity (g/L.h)	0.2305	0.2066
PHB Productivity (g/L.h)	0.1324	0.1134
g DCW/g Substrate	0.3688	0.3305
g PHB/g Substrate	0.2118	0.1815

## Data Availability

Data will be made available on request. The data presented in this study are available on request from the corresponding author.

## Supplementary Materials

The following supporting information can be downloaded at https://www.mdpi.com/article/10.3390/polym17030324/s1: Figure S1: Pareto chart of adjusted DCW model (soybean oil); Figure S2: Pareto chart of adjusted PHB accumulation model (soybean oil); Figure S3: Pareto chart of adjusted PHB production model (soybean oil); Figure S4: Pareto chart of adjusted DCW model (corn oil); Figure S5: Pareto chart of adjusted PHB accumulation model (corn oil); Figure S6: Pareto chart of adjusted PHB production model (corn oil).
