# Supplementary material for: Corn or Soybean Oil as the Sole Carbon Source for Polyhydroxybutyrate Production in a Biofuel Biorefinery Concept
Source: Polymers (Basel). 2025 Jan 25;17(3):324. doi: 10.3390/polym17030324 (PMC11821013; doi:10.3390/polym17030324)
Supplement: Supplementary file 1 [file polymers-17-00324-s001.zip › polymers-3396855-supplementary.pdf]

Corn and soybean oil as the sole carbon source for polyhydroxybutyrate production in a biofuel biorefinery concept

Clara Matte Borges Machado[a], Luciana Porto de Souza Vandenberghe[a] *, Ariane Fátima Murawski de Mello[a], Carlos Ricardo Soccol[a].

[a] Federal University of Paraná, Department of Bioprocess Engineering and Biotechnology, Centro Politécnico, 81531-980 Curitiba, Paraná, Brazil.

*Corresponding author: lvandenberghe@ufpr.br

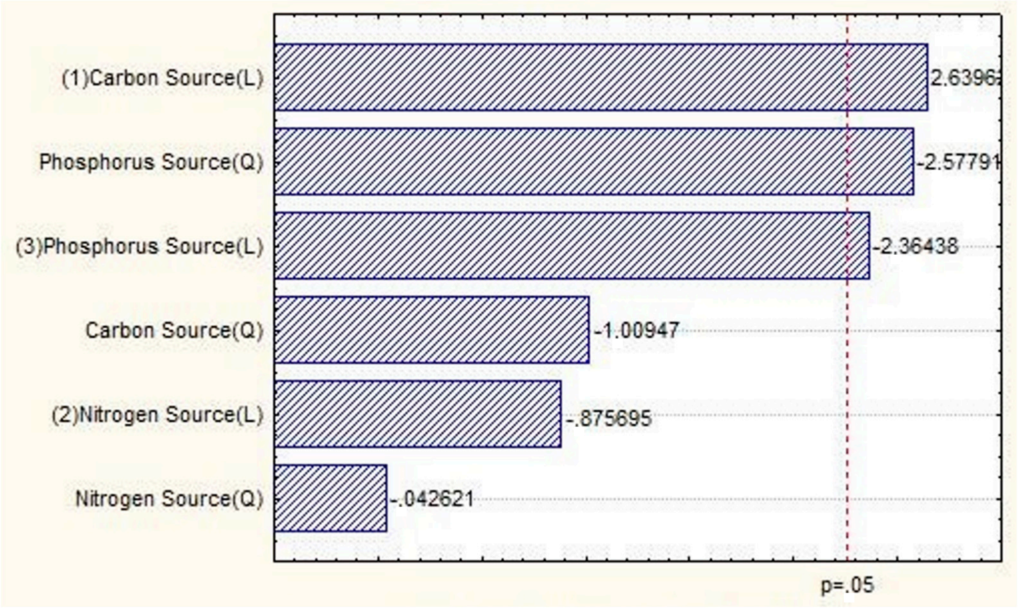

Fig S1. Pareto Chart of adjusted DCW model (soybean oil). Values ranging from 0.9 to 0.0 had their first significant algorithm suppressed (i.e. the 0 before the point).

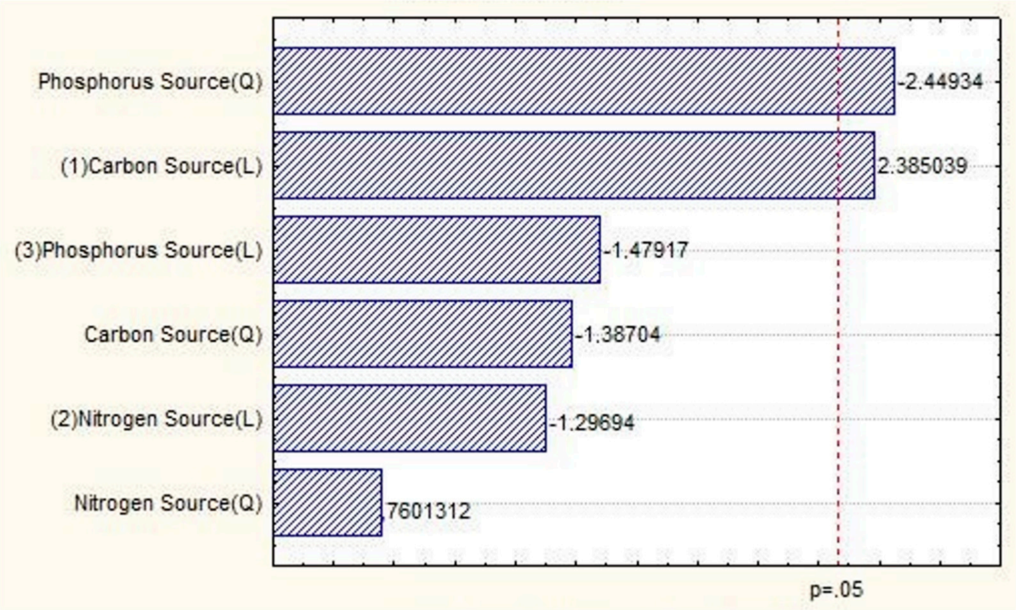

Fig S2. Pareto Chart of adjusted PHB accumulation model (soybean oil). Values ranging from 0.9 to 0.0 had their first significant algorithm suppressed (i.e. the 0 before the point).

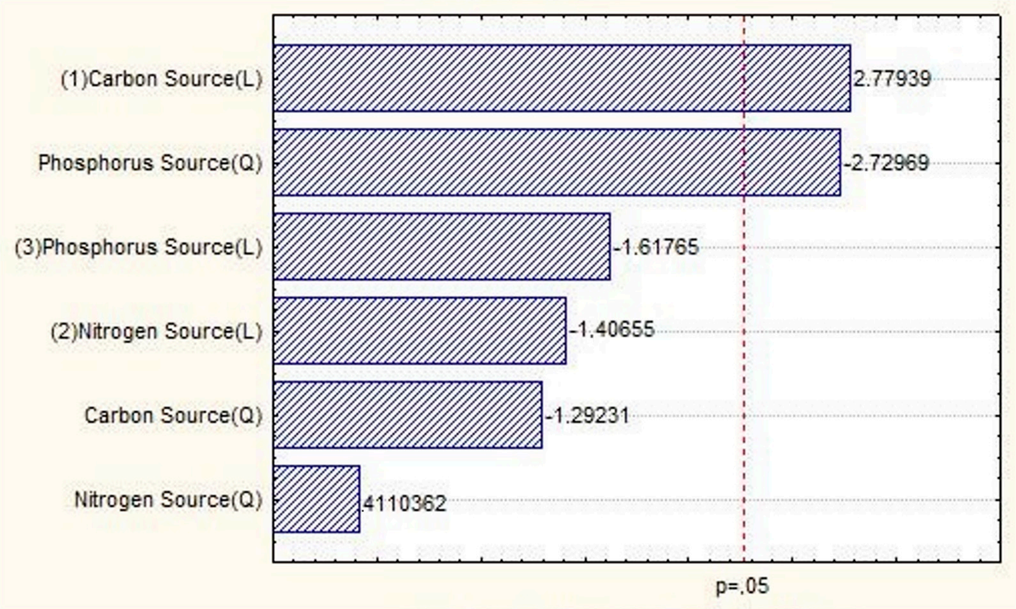

Fig S3. Pareto Chart of adjusted PHB production model (soybean oil). Values ranging from 0.9 to 0.0 had their first significant algorithm suppressed (i.e. the 0 before the point).

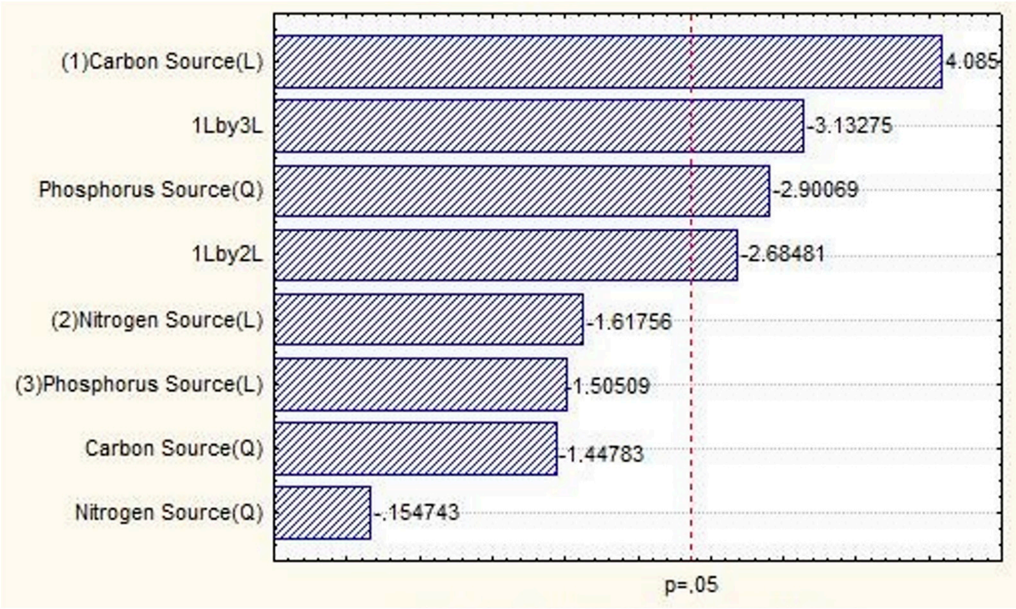

Fig S4. Pareto Chart of adjusted DCW model (corn oil). Values ranging from 0.9 to 0.0 had their first significant algorithm suppressed (i.e. the 0 before the point).

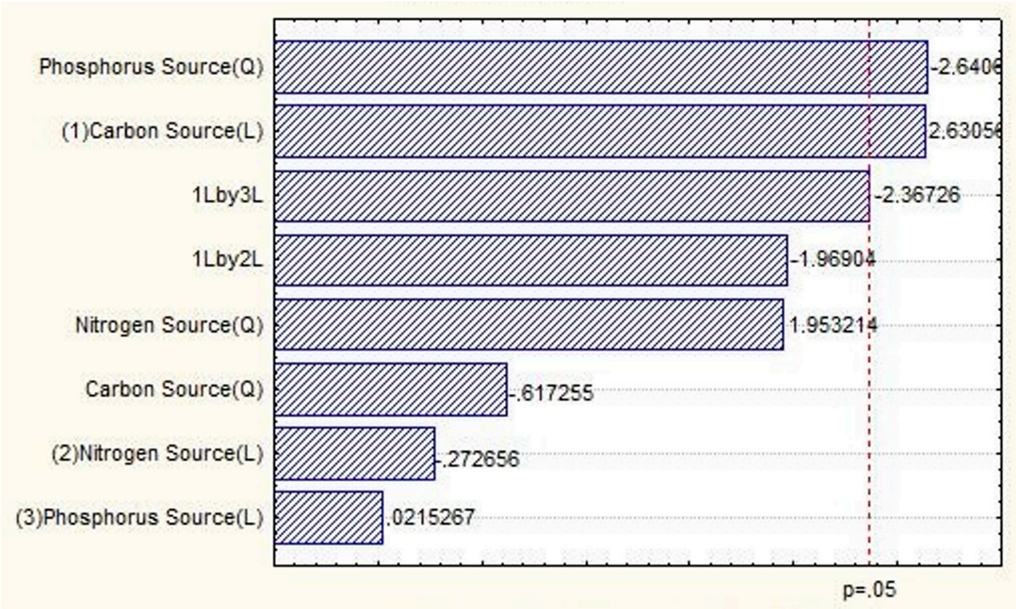

Fig S5. Pareto Chart of adjusted PHB accumulation model (corn oil). Values ranging from 0.9 to 0.0 had their first significant algorithm suppressed (i.e. the 0 before the point).

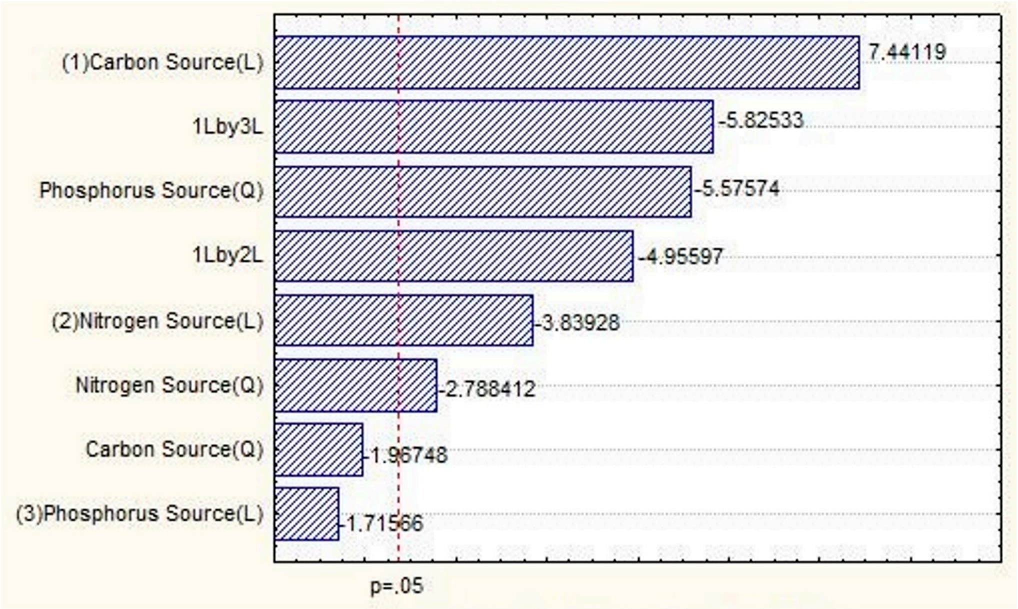

Fig S6. Pareto Chart of adjusted PHB production model (corn oil). Values ranging from 0.9 to 0.0 had their first significant algorithm suppressed (i.e. the 0 before the point).
